# Effect of Superheated Steam Treatment on the Mechanical Properties and Dimensional Stability of PALF/PLA Biocomposite

**DOI:** 10.3390/polym11030482

**Published:** 2019-03-13

**Authors:** Ahmed Jaafar Hussein Challabi, Buong Woei Chieng, Nor Azowa Ibrahim, Hidayah Ariffin, Norhazlin Zainuddin

**Affiliations:** 1 Department of Chemistry, Faculty of Science, Universiti Putra Malaysia, 43400 UPM Serdang, Selangor, Malaysia; ahmed.challabi@yahoo.com (A.J.H.C.); norhazlin@upm.edu.my (N.Z.); 2 Materials Processing and Technology Laboratory, Institute of Advanced Technology, Universiti Putra Malaysia, 43400 UPM Serdang, Selangor, Malaysia; 3 Department of Bioprocess Technology, Faculty of Biotechnology and Biomolecular Sciences, Universiti Putra Malaysia, 43400 UPM Serdang, Selangor, Malaysia; hidayah@upm.edu.my

**Keywords:** superheated steam, pineapple leaf, biocomposite

## Abstract

The effectiveness of superheated steam (SHS) as an alternative, eco-friendly treatment method to modify the surface of pineapple leaf fiber (PALF) for biocomposite applications was investigated. The aim of this treatment was to improve the interfacial adhesion between the fiber and the polymer. The treatment was carried out in an SHS oven for different temperatures (190–230 °C) and times (30–120 min). Biocomposites fabricated from SHS-treated PALFs and polylactic acid (PLA) at a weight ratio of 30:70 were prepared via melt-blending techniques. The mechanical properties, dimensional stability, scanning electron microscopy (SEM), and X-ray diffraction (XRD) for the biocomposites were evaluated. Results showed that treatment at temperature of 220 °C for 60 min gave the optimum tensile properties compared to other treatment temperatures. The tensile, flexural, and impact properties as well as the dimensional stability of the biocomposites were enhanced by the presence of SHS-treated PALF. The SEM analysis showed improvement in the interfacial adhesion between PLA and SHS-treated PALF. XRD analysis showed an increase in the crystallinity with the addition of SHS-PALF. The results suggest that SHS can be used as an environmentally friendly treatment method for the modification of PALF in biocomposite production.

## 1. Introduction

There has recently been growing interest in natural fibers due to their ability to reinforce polymers and replace synthetic fibers in the fabrication of composites or biocomposites. The widespread application of natural fibers can be attributed to their many advantages, such as being environmentally friendly, low cost, nontoxic, renewable, and having high strength. Natural fibers, such as hemp, flax, jute, oil palm, and kenaf, have been utilized in a variety of composite applications, including automotive, construction, and aerospace [1,2]. Another study reported the incorporation of natural fiber composites in the car industry for both interior and exterior car parts [3].

Malaysia ranks ninth in the world among the exporter countries for pineapple, with 425,019.75 metric ton of total production annually [4]. Pineapple leaf fibers (PALF) are one of the waste materials in the agriculture sector, with very large production in Malaysia as well as in Asia. Pineapple leaf fibers have been used to reinforce polymers in composite fabrication due to their high strength and good mechanical properties compared to other natural fibers [5]. Moreover, pineapple leaf fibers contain high amounts of cellulose, which may attribute to their good mechanical properties. The chemical composition of pineapple leaf fibers was reported by Jose et al. [6] as 80%–81% cellulose, 16%–19% hemicellulose, and 4.6%–12% lignin. In addition, pineapple leaf fibers have been used to reinforce polymers in biocomposite fabrication and shown to make a significant improvement in the mechanical and thermal properties of the biocomposites [7,8].

Polylactic acid (PLA) is a biodegradable thermoplastic polymer that comes from renewable natural resources, such as corn starch and sugar cane. The two most common methods for PLA synthesis are ring-opening polymerization and condensation [9]. PLA is used in a variety of applications due to its good mechanical properties, good biodegradability, and biocompatibility [10]. However, PLA still has some drawbacks, such as high cost, brittleness, and poor toughness. Attempts to overcome these issues have included blending PLA with other polymers, modifying PLA with plasticizer, and blending PLA with inorganic nanofillers [11].

In a previous study, PALF was used to reinforce PLA via melt-blending techniques [12]. However, there were some drawbacks due to poor interfacial adhesion between the fiber and the matrix resulting from the hydrophilic nature of PALF and the hydrophobicity nature of PLA. Thus, attempts have been made to modify the fiber surface in order to improve the interfacial adhesion. Most of these modification methods used chemical treatment, such as alkali with NaOH [13,14,15], bleaching [9], chemical grafting [16], or silane [17]. All these modification methods improved the biocomposite properties, but most of the methods are not eco-friendly as they use chemicals or are toxic and expensive. Therefore, there is still a need for a cheap and environmentally friendly modification method.

Superheated steam (SHS) has recently been used as an alternative to other chemical treatment methods. SHS is a steam produced by boiling wet steam with additional heat at a given pressure, which can be used in drying processes [18]. It is a cheap and eco-friendly treatment for surface modification of fibers because as water is the only element used in this technique [19]. Thus, it can be a cost-effective method with low risk. Superheated steam has been applied for lignocellulosic materials in various applications, for example, biofuel production, activated carbon, and bioabsorbents among others [20]. Furthermore, studies have shown the ability of SHS treatment to remove hemicellulose particles, which can attribute to the increase in cellulose and lignin content in fibers [21]. Another study by Hosseinaei et al. [22] showed an increase in fiber hydrophobicity and thermal stability attributed to the removal of hemicellulose. All the above properties can be advantageous in biocomposite production. For this reason, superheated steam is a promising alternative treatment method for the modification of fibers in biocomposite fabrication. To the best of our knowledge, there has not been any previous study on the utilization of SHS to modify PALF.

Based on the described issues, the main objective of this work was the modification of PALF surface. Fourier transform infrared (FTIR) spectroscopy and scanning electron microscopy (SEM) were used for the characterization of the treated fiber. The effect of SHS-treated PALF on the mechanical properties and dimensional stability of PALF/PLA biocomposites were then examined.

## 2. Materials and Methods 

### 2.1. Materials

PLA was purchased from Natureworks LLC (Minnetinka, MN, USA) under the trade name polylactide resin 3052D. It has a density of 1.24 g/cm^3^, melting point range between 170 and 190 °C, and a molecular weight of 93,500 g/mol. PALF was procured from Nature Renascent (Johor Bahru, Malaysia). It was supplied in bundles of long fibers, which was then dried under sunlight and ground, sieved into sizes of 300–500 μm, and kept in sealed plastic bags. Table 1 present the chemical composition of PALF.

### 2.2. Modification of PALF by SHS Treatment

Modification of PALF by SHS was carried out in a superheated steam oven (Model QF-5200C, Naomoto Corporation, Osaka, Japan) under normal pressure following the methods described by Then et al. [19] and Nordin et al. [23]. Tap water was used to produce SHS. PALF was dried in an oven at 60 °C before the treatment. The dried PALF was then treated with SHS oven at temperature of 190, 200, 210, 220, and 230 °C for 60 min. The experiment procedure was as follows. First, the SHS oven was turned on and allowed to reach a steady state of the desired condition. Next, PALF was spread on an aluminum foil tray. It was then put into the heating chamber of the SHS oven under the set condition. Once the treatment was complete, the fiber was removed immediately from the heating chamber and cooled down in a desiccator before being placed in a sealed plastic bag. The PALF treated at 220 °C showed the best tensile results for the biocomposite compared to other treatment temperatures. Therefore, it was chosen to conduct further experiment with the same treatment procedures at different treatment times (30, 90, and 120 min) to determine the best treatment time.

### 2.3. Fabrication of Biocomposites

To prepare a biocomposites, the oven-dried PALF, together with PLA, was prepared by the melt-blending technique using Brabender Internal Mixer at 160 °C with 50 rpm of rotor speed and for 15 min, as the method described by Birnin-Yauri et al. [24]. First, the PLA pellets were put into the mixing chamber to melt for 2 min. Then, the fibers were added, and the blending was continued for 13 min, followed by compression molding to produce sheets with thickness of 1 mm and 3 mm. Compression molding was performed using a hydraulic hot press at 160 °C molding temperature, 150 kg/cm^2^ pressure, and for 5 min. After that, cooling was performed at 30 °C for 5 min.

### 2.4. Characterizations

#### 2.4.1. Fourier Transform Infrared (FTIR) Spectroscopy

The functional groups and the chemical components of the untreated PALF and SHS-PALF were identified by FTIR using Perkin Elmer Model 100 series (Waltham, MA, USA) equipped with attenuated total reflectance (ATR). The FTIR spectra of the samples were recorded in the wavenumber ranging 400–4000 cm^−1^. 

#### 2.4.2. Mechanical Properties

A tensile property test was performed using Universal Testing Machine Instron Model 5566. Five specimens were tested following the ASTM D638 (type V). The test was conducted at room temperature with 1 kN load cell and a constant crosshead speed of 10 mm/min. The tensile strength, tensile modulus, and elongation at break were obtained from the test. 

A three-point bending test was conducted on the biocomposites using Universal Testing Machine Instron Model 5566. Five specimens were tested using ASTM D790, with the test conducted at 25 °C with 1 kN load. The results were expressed in terms of flexural strength and flexural modulus. 

The impact strength of the biocomposites was tested using an un-notched Izod impact test. The impact tester (Mumbai, India) was equipped with a 7.5 J pendulum. The test was conducted at 25 °C, and five specimens were tested using the ASTM D256 with dimension size 63.5 × 12.7 × 3.0 mm^3^. The impact strength (J/m) was calculated by dividing the energy (J) obtained with the thickness (m) of the specimen.

#### 2.4.3. Scanning Electron Microscopy

The scanning electron micrographs of the tensile fracture surface of PALF and SHS-PALF were recorded using a JEOL (Tokyo, Japan) JSM-6400 scanning electron microscope operated at 15 kV accelerating voltage. The samples were first oven-dried, then put on a metal holder and coated with gold using a Bio-rad (Hercules, CA, USA) coating system for 3 min to ensure better conductivity prior to analysis.

#### 2.4.4. X-Ray Diffraction (XRD) Analysis

The crystallinity of neat PLA and PALF/PLA and SHS-PALF/PLA biocomposites were analyzed using a Shimadzu XRD 600 diffractometer (Tokyo, Japan) with a nickel-filtered Cu Kα (λ = 0.1542 nm) beam performed at 30 kV and 30 mA. The samples were examined with a scanning rate of 2°/min at 25 °C within a 2θ range of 10° to 60°.

#### 2.4.5. Dimensional Stability Measurement

Water uptake and thickness swelling of the biocomposites were determined according to the ASTM D570 and European Standard EN 317 (1993), respectively. Samples with dimension of 10.0 × 10.0 × 1.0 mm^3^ were cut from the sample sheets and used for testing. Prior to the testing, the samples were dried in an oven at 60 °C until a constant weight was obtained. The initial weight (W_0_) and thickness (T_0_) of the dried samples were measured, and the samples were then soaked in distilled water for 24 h at 25 °C. After that, the samples were removed and wiped with tissue paper to remove excess water on the surface of the samples. The final weight (W_24h_) and thickness (T_24h_) of the samples were measured immediately. Five specimens were tested, and the average value and standard deviation were calculated. The water uptake and thickness swelling of the biocomposites were calculated based on Equations (1) and (2), respectively:

(1) $$\mathrm{Water}\;\mathrm{uptake}\;(\%)\;=\;\frac{w_{24h}-w_{0}}{w_{0}}\times 100$$

(2) $$\mathrm{Thickness}\;\mathrm{swelling}\;(\%)\;=\;\frac{T_{24h}-T_{0}}{T_{0}}\times 100$$

## 3. Results and Discussion

### 3.1. Characterization of PALF and SHS-PALF

#### 3.1.1. Fourier Transform Infrared (FTIR) Analysis

**Figure 1:**
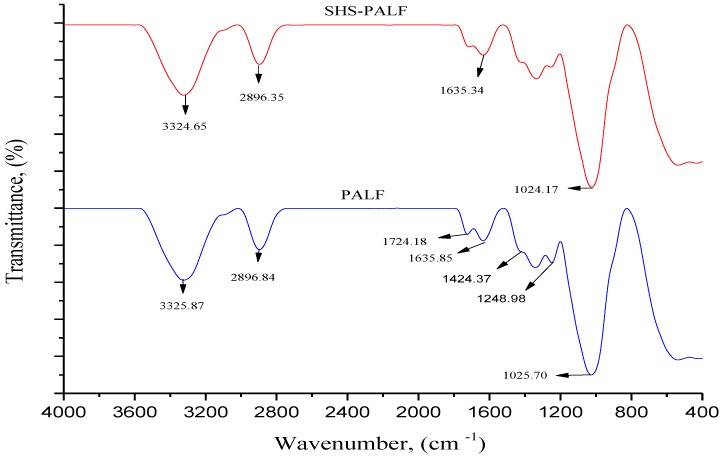
FTIR spectra of PALF and superheated steam-treated PALF (SHS-PALF).

The FTIR was used to locate and observe the functional group and chemical components of the fiber. The FTIR spectra in Figure 1 represent the untreated PALF and SHS-treated PALF with treatment temperature of 220 °C for 60 min, which was shown to have the best tensile properties for the biocomposite (discussed in Section 3.2.1). The most significant difference that can be seen in the FTIR spectra is the peak at 1724 cm^−1^, which corresponds to the acetyl group and C=O bond and represents the hemicellulose characterization. The absence of this peak for SHS-treated fiber can also be observed, indicating the removal of the hemicellulose for the SHS-treated PALF. A similar observation was reported by Sena Neto et al. [25]. In addition, a reduction occurred in the hydrophilicity of SHS-PALF in comparison to the untreated PALF, which was confirmed by the decrease in the absorbance of peak at 1632 cm^−1^, which corresponds to OH stretching of absorbed water [26]. Moreover, there was an absence of the peak at 1248.98 cm^−1^ in the case of SHS-PALF. This peak is attributed to the C–O stretching in acetyl in xylan, and the absence was due to the removal of the acetyl groups presented in the hemicellulose [27].

#### 3.1.2. Surface Morphology

**Figure 2:**
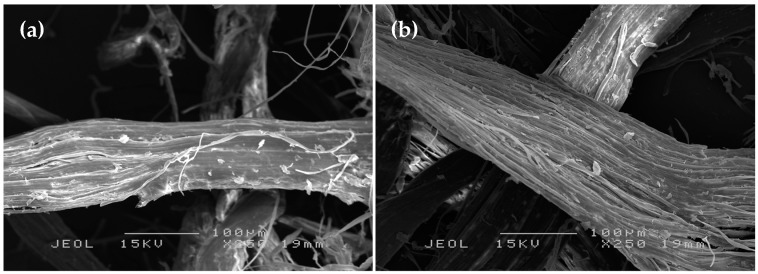
SEM micrographs of (**a**) 250x PALF and (**b**) 250x SHS-PALF.

Figure 2 presents the surface morphology from the SEM analysis for the untreated PALF and SHS-treated PALF with treatment temperature of 220 °C for 60 min, which was has shown to have the best tensile properties for the biocomposite (discussed in Section 3.2.1). The untreated PALF, shown in Figure 2a, had a smooth surface covered with impurities and some waxy substances. A similar observation was reported by Dong et al. [28]. However, after SHS treatment, it could be observed that the treated PALF, shown in Figure 2b, did not have these impurities, and the fiber surface became rougher. A similar observation was reported by Panyasart et al. [29].

The SEM and FTIR analyses demonstrated the ability of SHS treatment to improve the fiber’s properties by removing the surface impurities and making the fiber surface rougher for better interfacial adhesion between the fiber and the polymer. A similar observation was reported for PALF treated with NaOH, which was shown to improve the properties of the fiber [13]. However, the SHS treatment can be classified as more environmentally friendly modification method in comparison to NaOH treatment as no chemicals are used during the process of fiber treatment. Moreover, the fiber that has been modified by SHS treatment can be utilized without any drying process for biocomposite fabrication. Thus, SHS can be used as an alternative to NaOH treatment for fiber modification.

### 3.2. Characterization of Untreated PALF/PLA and SHS-PALF/PLA Biocomposites

#### 3.2.1. Tensile Properties

In this work, PLA was used as the polymer matrix due to its good mechanical and environmental properties. PLA has been previously reported by Birnin-Yauri et al. [30] to have high strength, modulus, and biocompatibility. The fiber–matrix ratio in the biocomposite is 30:70 according to previous studies done on PALF biocomposites [31]. 

SHS was used as a treatment method to improve the surface adhesion between PLA and PALF. The effectiveness of SHS was evaluated by comparing the tensile results for the untreated fiber and treated fiber. Table 2 presents the results of tensile strength (TS), elongation at break (EB), and tensile modulus (TM) for the biocomposites. The neat PLA results showed a reduction in the mechanical properties after adding the natural fibers.

As can be seen from Table 2, at the treatment time of 60 min and treatment temperature of 190 °C, the TS and EB for treated PALF remained almost the same as the untreated PALF, while there was an improvement in TM of 13%. The increase in TS, EB, and TM occurred with the increase in the treatment temperature from 200 to 220 °C and decreased at 230 °C. At 220 °C, the improvement in TS and EB were recorded as 24% and 14%, respectively, compared to the untreated PALF. The TM increased with the increase in treatment temperature up to 220 °C, reached the highest value at 220 °C for 120 min, then decreased at 230 °C. At 220 °C for 60 min, the increase in TM was 16% in comparison to that of the untreated PALF. It is obvious from the table that the TS was optimum at 220 °C for 60 min, as were the EB and the TM, in comparison to the untreated PALF/PLA. Thus, this temperature was selected to conduct further experiments in order to determine the optimum time for this treatment. 

**Figure 3:**
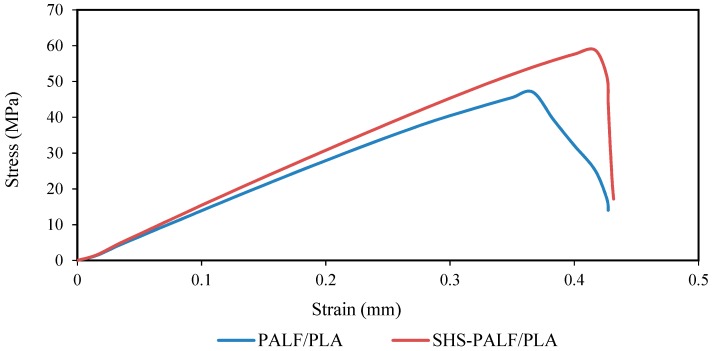
Typical tensile curves for PALF/PLA and SHS-PALF/PLA biocomposites.

Figure 3 shows typical tensile curves of PALF/PLA and SHS-PALF/PLA biocomposites with treatment condition of 220 °C and 60 min. The figure clearly shows that both tensile stress and elongation improved for the SHS-PALF/PLA biocomposite compared to the PALF/PLA one and that the SHS-PALF/PLA biocomposite had slightly better stiffness compared to the PALF/PLA biocomposite.

The TS and EB increased at 60 min treatment time compared to other treatment times, but decreased and remained stable for other treatment times (30, 90, 120 min). However, TM was also stable from 60 to 90 min, and it reached its highest value at 120 min with 30% improvement compared to the untreated PALF and 12% improvement compared to 60 min treatment time. From the tensile results, it could be concluded that treated PALF at 220 °C and 60 min gave the best biocomposite with the optimum TS, EB, and TM compared to the untreated PALF as well as to other treatment temperatures and times. 

As can be seen, SHS treatment improved the tensile properties in the PALF/PLA biocomposite. This might have been due to the removal of impurities on the fiber surface in the case of SHS-PALF, as shown in Section 3.1.2, which led to a rough fiber surface, thus promoting a better interfacial bonding and additional improvement in mechanical interlocking between the matrix and fibers [32]. Moreover, there was a gradual increase in the fiber hydrophobicity of SHS-PALF from 200 to 220 °C due to the reduction in water absorption of the fiber [33]. This might have resulted in better compatibility with the hydrophobic PLA, which improved the tensile properties of the biocomposite. As has been previously reported, SHS treatment works by removing the hemicellulose from the fiber and changing the chemical compound of the fiber by increasing the cellulose and lignin content [23]. It was observed that, at 200 to 220 °C treatment temperature, there was an enhancement in the tensile strength with the removal of hemicellulose from the fiber. This led to an improvement in the mechanical performance of the biocomposite [34]. Moreover, the best value for elongation at break was at 220 °C and 60 min, indicating that, the interfacial bonding of the SHS-PALF/PLA biocomposite was strong at this treatment temperature. A similar observation can be made for the reduction of tensile strength with high treatment temperature of 230 °C. Here, the degradation of hemicellulose would occur rapidly by liberation of large amount of acetic acid [19]. This may accumulate on the fiber surface and accelerate the degradation process of cellulose. Thus, it will affect the mechanical strength of the biocomposite.

#### 3.2.2. Fracture Surface Morphology

**Figure 4:**
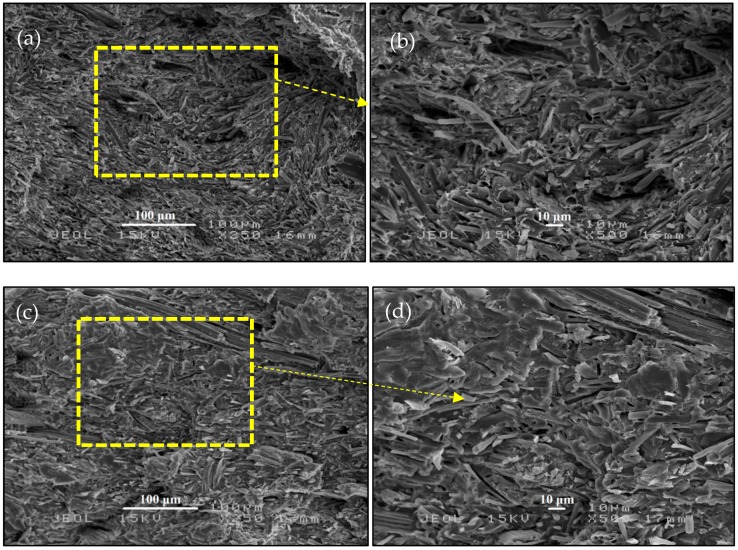
SEM micrographs of PALF/PLA (**a**) 250x, (**b**) 500x; and SHS-PALF/PLA (**c**) 250x, (**d**) 500x biocomposites.

The tensile fracture surface of PALF/PLA and SHS-PLAF/PLA with treatment temperature of 220 °C and 60 min were examined under scanning electron microscope in order to study the adhesion between the fiber and the matrix. As shown in Figure 4a,b, the untreated PALF and PLA had poor interfacial adhesion due to the incompatibility between the hydrophilic PALF and the hydrophobic PLA. Furthermore, the voids were visible in the structure of the biocomposite, and those voids between the two phases led to poor adhesion and decrease in properties [35]. However, the morphology for the SHS-PALF/PLA biocomposite, given in Figure 4c,d, showed a better interfacial adhesion compared to the untreated PALF/PLA biocomposite and looked solid in structure. Moreover, there were no visible voids on the surface of the biocomposite, with rougher fiber surface, which led to a better compatibility between PALF and PLA [36]. 

Additionally, it has been reported that lignin softens within a temperature range of 160–190 °C [19]; below this temperature, lignin is in solid phase. When lignin softens, it turns sticky and has adhesive force, which may attribute to spreading the lignin on the fiber surface. It then turns back to its solid state upon cooling [37]. Another factor is the fact that some of the hemicellulose elements may also be present on the fiber surface, and it may work on binding the fiber together with PLA during compounding. This would explain the good formation structure in the tensile fracture surface of the SHS-PALF/PLA biocomposite and the improvement in the properties of the SHS-PALF/PLA biocomposite.

#### 3.2.3. Flexural and Impact Properties

The results of flexural and impact tests on SHS-PALF/PLA biocomposite and PALF/PLA biocomposite with treatment temperature of 220 ℃ and time of 60 min are shown in Table 3. It can be seen that the flexural strength, flexural modulus, and impact strength for SHS-treated biocomposite had a higher value than the untreated biocomposite. The improvement in flexural strength, flexural modulus, and impact strength was 12%, 7%, and 16%, respectively. The increase in flexural and impact properties of SHS-PALF/PLA biocomposite resulted from the improvement in the interfacial adhesion between the fiber and the matrix. Moreover, a rough fiber surface led to a better fiber–matrix interlocking and thus an enhancement in the mechanical performance of the SHS-PALF/PLA biocomposite.

#### 3.2.4. Dimensional Stability

**Figure 5:**
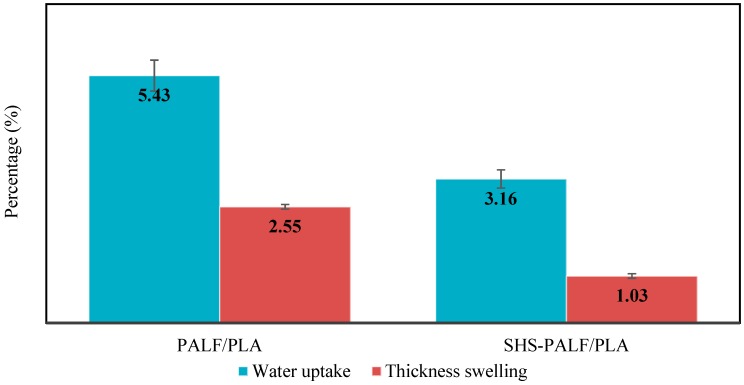
The water uptake and thickness swelling of PALF/PLA and SHS-PALF/PLA biocomposites.

One of the major drawbacks in natural fiber composites is the hydrophilic nature of the natural fiber [38]. The tendency of natural fibers to absorb moisture and swell when exposed to water or humid conditions limits the application of biocomposites. Therefore, water uptake and thickness swelling experiments are very important parameters to study when working with biocomposites. Figure 5 presents the water uptake and thickness swelling of PALF/PLA and SHS-PALF/PLA biocomposites with treatment temperature of 220 °C for 60 min after 24 h of immersion in water. Both biocomposites showed an increase in water uptake and thickness swelling due to the presence of hydroxyl groups in the fibers, which increased the amount of water absorbed into the composite [39]. However, the SHS-PALF/PLA biocomposite showed lower percentage of water uptake and thickness swelling compared to the untreated PALF/PLA biocomposite. 

As can be seen in Figure 5, the water uptake percentage of the SHS-PALF/PLA biocomposite decreased by 42% compared to the untreated PALF/PLA biocomposite. This result can be attributed to the removal of hemicellulose and the increase in cellulose content in the fiber as well as the decrement in the free space due to the improvement of the binding force. A similar observation was reported on the decrement of water uptake for the treated composite after alkali treatment [40]. Moreover, in this work, the water uptake of the biocomposite was not high. This can be attributed to the low fiber content in the biocomposite, where it contains 30 wt % of PALF. According to Muñoz et al. [41], the water uptake increases with the increase in fiber loading in the composite. The thickness swelling for the untreated PALF/PLA showed a reduction of 60% compared to SHS-PALF/PLA. This can be attributed to the low water uptake of the SHS-PALF/PLA biocomposite as the thickness swelling directly corresponds to the amount of water absorbed by the biocomposite.

#### 3.2.5. X-Ray Diffraction (XRD)

**Figure 6:**
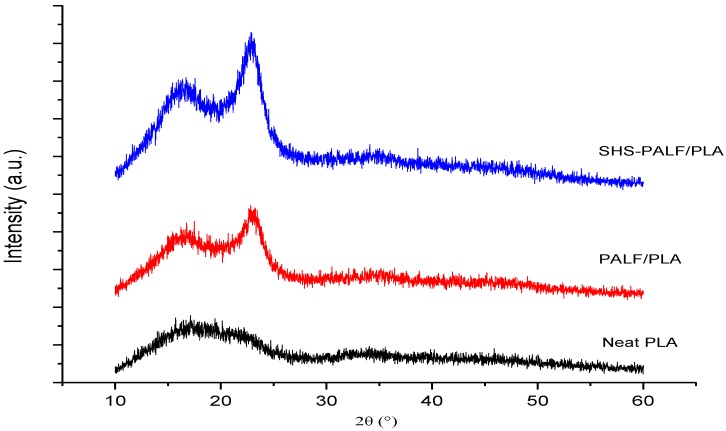
X-ray Diffraction (XRD) patterns of neat PLA, PALF/PLA, and SHS-PALF/PLA biocomposites.

The neat PLA, untreated PALF/PLA, and SHS-PALF/PLA biocomposites were characterized by X-ray diffraction to study the effect of the untreated PALF and SHS-PALF on the neat PLA crystallinity. As shown in Figure 6, the XRD patterns for neat PLA exhibited intensity, with a broad diffraction peak appearing at approximately 2θ ≈ 17°. The neat PLA did not show any characteristic peak, indicating that PLA has an amorphous structure [42]. In the case of PALF/PLA and SHS-PALF/PLA biocomposites, it can be observed that the XRD patterns exhibited two diffraction peaks at approximately 2θ ≈ 16° and 2θ ≈ 22°. These two peaks can be indexed to the native cellulose crystalline structure (cellulose I), similar to the study reported by Sena Neto [25]. The presence of these two diffraction peaks can be clearly attributed to the existence of PALF in the biocomposite. In addition, the diffraction patterns of the SHS-PALF/PLA biocomposite was similar to the PALF/PLA biocomposite, indicating that the SHS treatment did not change the cellulose’s crystal structure [15]. Furthermore, the intensity of the diffraction peak became stronger, confirming the increase in the crystallinity of the SHS-PALF/PLA biocomposite [9]. This result supports the improvement in the mechanical performance of the SHS-PALF/PLA biocomposite. As reported by Mathew et al. [43], higher crystallinity of composites is considered as one of the factors for better mechanical performance.

## 4. Conclusions

In the present work, SHS was successfully used as a treatment method to modify the surface of PALF. An improvement in the roughness of the fiber surface was achieved, which can be attributed to the removal of hemicellulose and impurities. The tensile properties showed the optimum result at 220 °C for 60 min treatment condition. It was found that the increase in tensile strength, tensile modulus, and elongation at break were 24%, 16%, and 14%, respectively. The SEM analysis showed improvement in the interfacial adhesion of the SHS-PALF/PLA biocomposite, while the XRD graph showed the increase in the crystallinity of the biocomposite after the SHS treatment. Moreover, the flexural strength, flexural modulus, and impact were also enhanced by 12%, 7%, and 16%, respectively, for the SHS-PALF/PLA biocomposite. Dimensional stability also showed improvement in water uptake and thickness swelling of 42% and 60%, respectively. This work therefore proves that SHS can be successfully used as an environmentally friendly processing method in modifying the PALF surface that can be an alternative to the conventional chemical methods that are utilized in biocomposite production. 

## Figures and Tables

**Table 1:** Chemical composition of the raw pineapple leaf fiber (PALF).

Fiber	Cellulose (%)	Hemicellulose (%)	Lignin (%)
PALF	69.89	19.67	10.43

**Table 2:** Mechanical properties of PALF/PLA and SHS-PALF/PLA biocomposites.

	Treatment Temperature °C	Treatment Time (min)	Tensile Strength (MPa)	Elongation at Break (%)	Tensile Modulus (GPa)
PLA	–	–	63.54 ± 0.11	6.64 ± 0.30	1.14 ± 0.05
PALF/PLA	–	–	46.82 ± 1.75	3.67 ± 0.17	1.39 ± 0.02
SHS-PALF/PLA	190	60	46.69 ± 1.13	3.55 ± 0.09	1.58 ± 0.07
	200	60	50.24 ± 1.40	3.84 ± 0.17	1.59 ± 0.06
	210	60	50.01 ± 1.13	3.73 ± 0.20	1.56 ± 0.03
	220	30	53.01 ± 1.33	3.75 ± 0.17	1.63 ± 0.05
		60	57.94 ± 1.51	4.18 ± 0.19	1.61 ± 0.03
		90	52.1 ± 1.60	3.85 ± 0.00	1.61 ± 0.04
		120	51.43 ± 2.14	3.67 ± 0.17	1.81 ± 0.09
	230	60	41.20 ± 1.32	2.97 ± 0.17	1.54 ± 0.01

**Table 3:** Flexural strength, flexural modulus, and impact strength of PALF/PLA and SHS-PALF/PLA biocomposites.

Biocomposite	Flexural Strength (MPa)	Flexural Modulus (GPa)	Impact Strength (J/m)
PALF/PLA	70.53 ± 1.52	5.2 ± 0.18	115.04 ± 3.95
SHS-PALF/PLA	79.27 ± 0.43	5.57 ± 0.16	133.73 ± 6.49
